# Programmable large-cargo integration: Overcoming size constraints for next-generation gene therapy

**DOI:** 10.1016/j.synbio.2025.11.008

**Published:** 2025-12-02

**Authors:** Lifang Yu, Mario Andrea Marchisio

**Affiliations:** a School of Chemistry and Chemical Engineering, Huangshan University, Huangshan, 245041, PR China; b School of Life and Health Science, Northeastern University, Shenyang, 110169, PR China

**Keywords:** Large DNA insertion, CASTs, PE-integrase, nCas9-R2, Clinical therapy

## Abstract

The emergence of base and prime editors—genome editing tools that avoid double-strand breaks (DSBs)—has enabled precise point mutations, insertions, inversions, deletions, and substitutions, which accelerates the development of single-intervention therapies and advances individualized genomic medicine. However, their limited efficiency in inserting large DNA fragments has restricted applications for correcting diverse pathogenic mutations within a single gene. In this review, we explore three recently developed strategies for efficient large DNA cargo insertion (>1 kb): CRISPR-associated Tn7-like transposases (CASTs), PE-integrase systems, and R2 retrotransposon fusions (nCas9-R2). We examine the applications of these systems in both bacterial and mammalian contexts and discuss their respective advantages and current limitations. Finally, we address persistent challenges and propose potential directions to guide future research.

## 1 Introduction

In recent decades, the emergence and development of CRISPR-Cas systems—the RNA-guided adaptive immune mechanisms found in bacteria and archaea—have provided powerful tools for programmable gene modification in living organisms [1,2]. The activity of CRISPR-Cas systems begins with a guide RNA (gRNA) forming a ribonucleoprotein (RNP) complex with the Cascade/Cas protein. This RNP complex then binds to the target DNA substrate after recognizing the protospacer adjacent motif (PAM) [3,4]. Subsequently, the Cas protein mediates the induction of double-strand breaks (DSBs) in the target DNA, which are repaired by either homologous recombination (HR) or non-homologous end-joining (NHEJ) [5,6].

In comparison to the first-generation gene editing tools, such as Zinc Finger Nucleases (ZFNs) and Transcription Activator-Like Effector Nucleases (TALENs) [7], the CRISPR-Cas systems—particularly the Type II CRISPR-Cas9 and Type V CRSIPR-Cas12a—offer significant advantages in efficiency, simplicity, and versatility. A fundamental distinction lies in their targeting mechanism: ZFNs and TALENs require the *de novo* design and synthesis of unique, custom protein pairs for each target sequence [8]. In contrast, Class II CRISPR-Cas systems employ a single, multifunctional Cas protein paired with a readily programmable gRNA [3,4,9]. This architecture permits rapid retargeting by modifying only the gRNA sequences, eliminating the requirement of protein re-engineering and enhancing editing efficiency with reduced complexity.

The natural CRISPR-Cas immune response comprises three distinct stages: 1) spacer acquisition (capturing fragments from foreign DNA); 2) CRISPR array expression and processing to generate mature gRNAs; and 3) target interference mediated by the RNP complex [3,4,10]. Focusing on the widely used CRISPR-Cas9 system, the maturation of the gRNA in stage 2 involves the coordinated action of RNase III and a trans-activating RNA (tracrRNA) [10]. Specifically, the mature functional complex is formed by hybridizing the CRISPR RNA (crRNA) with the tracrRNA, which is bypassed in engineered systems by designing a single guide RNA (sgRNA) [11]. DNA cleavage by Cas9 is executed by its two distinct catalytic domains: the HNH domain, which cleaves the target DNA strand (TS), and the RuvC domain, which cleaves the non-target strand (NTS) [4,12]. Conversely, Cas12a utilizes a single RuvC domain to cleave both DNA strands [13]. This structural divergence between Cas9 and Cas12a underpins their distinct catalytic mechanisms and influences their respective editing outcomes [14,15].

Despite the widespread use of CRISPR-Cas systems for genome editing in both eukaryotes and prokaryotes, the editing efficiency of CRISPR-Cas remains variable. This variability is heavily dependent on cell division and cell types, and the introduction of DSBs can result in uncontrollable insertions and deletions (indels) that are cytotoxic and present significant challenges for clinical applications [[16], [17], [18], [19]]. To address these limitations, recent advancements have led to the development of two CRISPR derivative systems—base and prime editors—by replacing the Cas9 protein with either a catalytically dead Cas9 (dCas9) or a Cas9 nickase (nCas9) to eliminate DSBs [20,21].

Base editing (BE) corrects point mutations in genomic DNA by fusing cytidine or adenine deaminases to a nCas9 variant (D10A), thus enabling precise nucleotide conversions without the requirement for DSBs [20,22]. Prime editors consist of two key components: prime editing (PE) guide RNA (pegRNA) and a nCas9-based fusion protein [21]. The pegRNA is a modified version of the guide RNA and includes three crucial regions: the gRNA sequence, the reverse transcription template (RTT), and the primer binding sequence (PBS). The nCas9 (H840A) protein in PE is fused with a reverse transcriptase (RT) domain [21]. This fusion protein enables RT-mediated DNA synthesis using the RTT sequence of the pegRNA as a template upon hybridization of the pegRNA's PBS to the target DNA [21]. BE/PE enables precise DNA point mutation, insertions, inversions, deletions, and substitutions, which provides great potential for single intervention therapies for different clinical diseases and progress in individualized genomic medicine [[23], [24], [25], [26]].

However, the clinical application of BE and PE is still limited by their dependency on mutation types. BE/PE requires precise matching to specific mutation types, such as single-nucleotide variants (SNVs) or small indels. This renders these technologies non-universal to address the diverse pathogenic mutations present within a single gene. For example, in cystic fibrosis (CF), over 2000 CF transmembrane conductance regulator (CFTR) pathogenic variants have been documented, with more than 700 mutants proven to cause CF [27]. Currently, conventional BE/PE approaches can only target a small subset of these variants, such as the G542R and G551D SNVs [28,29]. They also exhibit limited efficiency against prevalent deletions like F508del, which accounts for 85 % of the global CF cases [27]. Diseases with highly heterogeneous mutational profiles—such as Duchenne muscular dystrophy (DMD) that involves large deletions (68 %), duplications (11 %), and small mutations (21 %) [30]—further highlight the limitations of BE/PE. These diseases necessitate the development of mutation-specific therapeutic strategies, which can drive up costs and limit the accessibility of the treatment. To overcome the constraints of gene-sized DNA sequence modifications, new large-fragment insertion technologies have emerged as a transformative solution. This approach involves the delivery of intact functional gene cassettes or gene clusters to either native or safe-harbor loci, bypassing the endogenous mutational mechanisms. Such strategies allow for single-intervention corrections of a wide array of variants in a target gene, providing a universal therapeutic solution for genetically heterogeneous disorders.

Ideally, a genome insertion technology for clinical applications requires the integration of gene-sized DNA cargo with high specificity and programmability, while circumventing the dependence on DSBs [31]. In this review, we first describe the development and characterization of three approaches—CRISPR-associated Tn7-like transposases (CASTs), PE-integrase, and R2 retrotransposon fusion (nCas9-R2)—used in large DNA (>1 kb) fragment integration, and then outline their applications in bacterial and mammalian cells.

## 2 CASTs

CASTs are chimeric genetic elements composed of two key components: (1) Tn7-like transposons, and (2) variants of CRISPR-Cas systems that are incomplete, after losing their canonical functions such as spacer acquisition and foreign DNA interference.

**Fig. 1:**
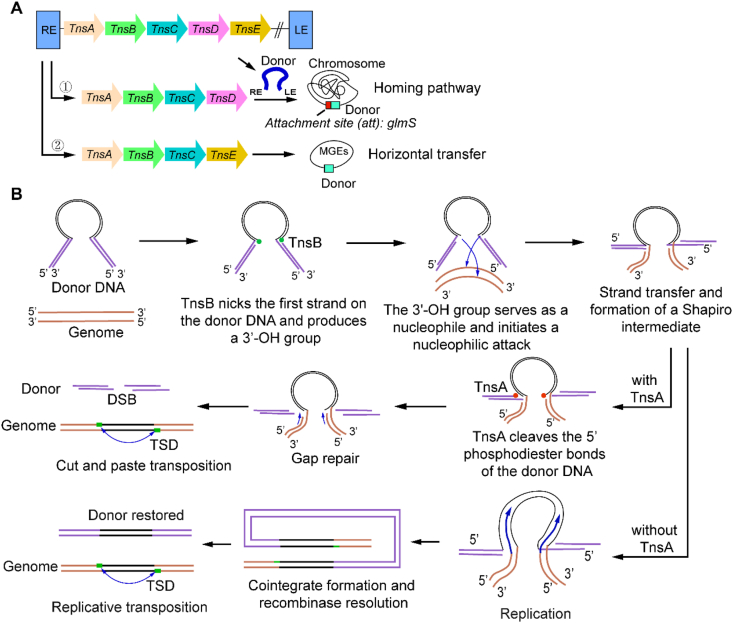
The transposition mechanism utilized by Tn7 and CASTs. (**A**) The two mutually exclusive transposition pathways of the Tn7 transposon. In pathway ①, the proteins TnsA, TnsB, TnsC, and TnsD collaborate to direct the precise insertion of the donor (here, the Tn7 transposon) into a highly conserved attachment site (e.g., *glmS*). Pathway ② shows TnsE-mediated DNA insertion into MGEs, which facilitates horizontal transfer, upon recognition of the replication intermediates. Double slashes (//) indicate omitted DNA sequences. (**B**) Roles of TnsA and TnsB in transposition [32]. TnsB first nicks one strand of the donor DNA, generating a 3′-OH group that serves as a nucleophile to attack the target DNA. This strand transfer event results in the formation of a Shapiro intermediate. In the presence of TnsA, the second strand of the donor DNA is cleaved, leading to the full release of the transposon. The excised donor DNA is then integrated into the genomic target site via the cellular gap repair mechanisms. In the absence of TnsA, the Shapiro intermediate undergoes replication, leading to the formation of cointegrates that are subsequently resolved by host recombinases. “TSD” means target site duplications.

Currently, all known CASTs are evolutionarily derived from the Tn7 transposon—the first bacterial transposon that was shown to target specific genomic loci [32,33]. In the Tn7 transposon, five genes—TnsA, TnsB, TnsC, TnsD and TnsE—are essential to carry out transposition [33]. TnsD and TnsE belong to two mutually exclusive transposition pathways: TnsD initiates conservative homing by binding conserved sites with its C-terminal DNA-binding domain, while TnsE mediates transposition to mobile genetic elements (MGEs) by sensing lagging-strand replication intermediates [33,34] (see Fig. 1A). The engagement with the target sequence triggers a hierarchical complex assembly: TnsD (through its N-terminal TniQ domain) or TnsE recruits the AAA^+^ ATPase TnsC, and ATP hydrolysis by TnsC drives conformational activation and formation of an active hexameric scaffold [35]. The activated TnsC complex recruits the TnsA-TnsB heteromeric transposase and places it at the target site [36]. During the DNA cleavage, TnsB selectively cuts the 3′ phosphodiester bonds at both the left (LE) and right (RE) ends of the transposon. TnsB also catalyzes 3′-end joining by attaching the 3′-OH groups of the transposon to the 5′-phosphates of the target DNA. This generates a Shapiro intermediate that bridges donor and target DNA. TnsA cleaves the 5′ phosphodiester bonds of the donor DNA, which releases the transposon and leaves a DSB in the donor molecule [32,36]. The genomic integration ends with the host-mediated gap repair: the cellular machinery fills the flanking gaps to create target site duplications (TSDs), which completes the non-replicative “cut-and-paste” transposition [37] (see Fig. 1B).

Bioinformatic analyses have currently categorized CASTs into six subtypes, five of which utilize a multi-subunit Class 1 CRISPR Cas system (types I–F, I–B, I-D, I–C, and IV), while the sole Class 2 representative (type V–K) employs a single-effector Cas12k protein [[38], [39], [40], [41], [42]]. To date, only a subset of these—including I–F, I–B, I-D, and V–K—have undergone extensive experimental characterization; the others rely primarily on computational predictions [31,41,[43], [44], [45]]. A key breakthrough was the engineering of the validated CAST systems (particularly I–F and V–K) to overcome their native insertion restrictions, which enabled RNA-directed integration of large genetic cargo (up to 10 kb) in heterologous hosts, including bacterial and mammalian cells [31,45,46]. These advances highlight the potential of CASTs as versatile tools for genome engineering. However, challenges in integration efficiency, specificity, and delivery must be addressed to have broader biological and therapeutic applications.

### 2.1 The discovery of CAST

The first CASTs, type I–F and I–B, were identified by a PSI-BLAST search (using Cas7f as a probe) and phylogenetic analysis. By leveraging the high conservation of Cas7f, the genes within 10 kb up- and downstream of each identified *cas7f* were annotated. This revealed that the vast majority of loci forming a distinct, strongly supported, monophyletic branch lacked *cas1* but contained a *tnsD/tniQ* (a homolog of *tnsD*) gene adjacent to the *cas* genes. Further phylogenetic analysis of both Cas7f and the associated TnsD/TniQ and TnsA transposon proteins demonstrated that the corresponding chimeric loci originated from distinct capture events: the *cas1*-less type I–F variant arose from a single ancestral capture event, which links it to a defined clade of Tn7-like transposons, whereas the type I–B system resulted from two small independent capture events with different transposon lineages [38,47].

Afterwards, the phylogenetic and genomic neighborhood analyses of 63 catalytically inactivated (mutations in the active site of the RuvC-like nuclease domain) CRISPR Type V–U5 effector proteins (later designated as V–K) from *cyanobacteria* revealed their consistent embedding within Tn7-like transposons. This association prompted the classification of V–K as a novel CAST subtype [40,44].

In 2021, a metagenomic mining study on 1476 CASTs led to the discovery of two novel CAST subtypes: Type I–C and Type IV. Their identification specifically required that: (1) systems must contain at least two Tn7-associated genes and three core Class 1 *cas* genes (*cas5/6/7/8*); and (2) any two *Tn7* genes must be ≤ 1500 bp apart, while any three core *cas* genes must be ≤ 1500 bp apart [39].

To further extend the subtypes of CAST, in 2023, Faure et al. employed an alternative strategy by using the conserved Tn7 component TnsC as a probe. The phylogenetic analysis of TnsC and its adjacent genes uncovered Type I-D CAST in the *cyanobacterium Cyanothece* sp. PCC 7425, which conclusively demonstrated its distinct evolutionary origin within the CAST family [42].

### 2.2 The general features of CASTs

Cas proteins and a CRISPR array, which are present in every canonical CRISPR-Cas system, are atypical components in CASTs. CASTs retain CRISPR-Cas activity only for crRNA processing and target recognition, thereby functioning as RNA-guided targeting complexes without executing DNA cleavage [47]. Among Type I CAST subtypes, all (except Type I-D) lack spacer acquisition and target cleavage capabilities because of the absence of *cas1*, *cas2*, and *cas3* genes [38,39,42]. In contrast, Type I-D CAST maintains functional spacer integration and cleavage activity, because it possesses intact *cas1* and *cas2* genes, and an active HD-nuclease domain within its Cas10d effector [42,[47], [48], [49]]. Type V–K CAST lacks *cas1* and *cas2* and differs from Type I systems for utilizing a single catalytically inactive Cas12k protein—instead of a multi-subunit Cascade complex—to guide transposition [40,44]. Compared to canonical CRISPR-Cas systems, whose CRISPR array embeds hundreds of repeats [[50], [51], [52]], the array in CASTs is significantly shorter, containing four repeats at most [41,43,44]. In some CASTs, like I–C and I–F, the array may lack spacers entirely or retain only a single self-target spacer [39,53,54].

Crucially, the absence of TnsA precludes a “cut-and-paste” transposition. In TnsA-deficient transposons, like V–K, transposition proceeds through replicative mechanisms that involve Shapiro intermediates and generate cointegrates, where the donor and the target DNA are fused [[55], [56], [57]] (see Fig. 1B). These cointegrates pose significant risks to host genomes by enabling chromosomal rearrangements [32]. While natural transposons (like Tn5053) often encode site-specific recombinases to resolve cointegrates, V–K CAST lacks both TnsA and a dedicated resolution machinery [57,58]. This deficiency is evidenced by the persistent cointegrate formation observed during V–K transposition in *Escherichia coli* [46,58] and represents a key limitation compared to Type I systems. To address it, Tou et al. engineered HELIX—a redesigned V–K CAST—by fusing the sequence-specific nickase nAnil to the N-terminus of TnsB. This artificial TnsA surrogate reduced cointegrate formation from 18.06 % (wild-type) to 0.49 % and restored precise “cut-and-paste” transposition effectively [46].

Tn7 and related bacterial transposons (e.g., Tn3 and Mu) exhibit target immunity—a mechanism preventing transposition into genomic sites already harboring cognate transposon sequences [[59], [60], [61]]. This process safeguards transposon structural integrity and enhances functional gene dissemination. Molecular studies confirmed that, in the Tn7 transposon, the interaction between TnsB, TnsC, and the terminal sequence of the transposon is essential for immunity. Indeed, TnsB can trigger ATP-dependent dissociation of TnsC from the transposon ends within the target DNA following integration [[62], [63], [64]]. Notably, this immunity extends to CAST [45,46]. Vo et al. (2021) observed that, in the engineered *Vibrio cholerae* I–F CAST (VcINT), donor insertion efficiency at targets with pre-existing Tn7 ends within 5 kb decreased to ∼20 % of that in non-immune controls. However, immunity dissipated when pre-integration distances exceeded 1 Mbp or when using orthogonal CAST systems (e.g., V–K subtypes) that lacked shared targeting determinants [45,65,66].

### 2.3 The two transposition pathways of CAST

CASTs repurpose CRISPR-Cas modules to execute RNA-programmed DNA integration, yet deploy two mechanistically independent transposition routes to bypass evolutionary constraints [41].

**Fig. 2:**
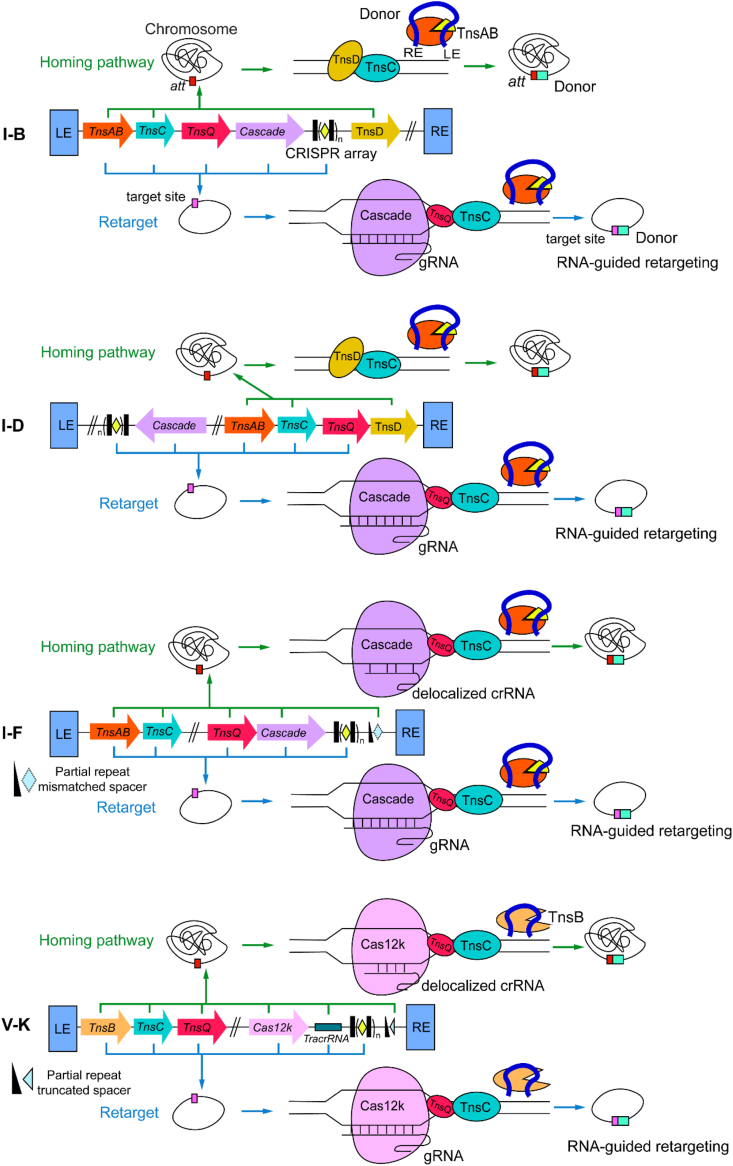
Transposition mechanisms across CAST subtypes [47]. Type I–B and I-D systems employ a TnsD-dependent pathway for homing and utilize the Cascade:gRNA complex for RNA-guided retargeting through spacer-mediated complementarity with the target DNA. In contrast, Type I–F and V–K CAST systems rely on a delocalized crRNA for homing. In Type I–F, the delocalized crRNA consists of a partial repeat and a mismatched spacer that anneals to sequences near the homing site. Type V–K systems utilize a gRNA with a truncated spacer that enables partial complementarity to the target region. For retargeting, Type I–F systems adapt the Cascade:gRNA complex, while Type V–K systems exploit an inactive Cas12k for RNA-guided DNA transposition. Attachment site abbreviated as “*att”*.

The homing pathway targets conserved genomic attachment sites (also termed homing sites, they are, for instance, loci adjacent to *glmS*, *tRNA*, or *parE* genes) to establish a stable chromosomal reservoir. This pathway circumvents lethal mutagenesis risks from random insertion and is mediated either by specialized TniQ-family proteins (e.g., TnsD) or crRNA variants, depending on the CAST subtype. For example, Type I–B1/I–B2/I-D/some I–F3 systems possessing dual TniQ-family proteins utilize dedicated TnsD for attachment site recognition and binding (I–B1 from *Anabaena variabilis* (hereafter AvCAST) is *glmS*-targeting; I–B2 from *Peltigera membranacea cyanobiont* 210A (PmcCAST) is *tRNA*^*Val*^-targeting; I-D from *Cyanothece* sp. PCC 7425 (CyCAST) is *tRNA*^*Leu*^-targeting; I–F3 from *Parashewanella curva* C51 (PcCAST) is *parE*-targeting [41,42,53,67,68]). Conversely, Type V–K and most I–F3 systems that lack TnsD employ delocalized crRNAs that locate outside of the CRISPR array but contain partial direct repeat (DR) and short spacers (e.g., V–K: 13-nt DR + 17-nt spacer targeting *tRNA*^*Leu*^) for homing (see Fig. 2) [41,65,69].

Concurrently, the dissemination pathway exploits RNA-guided targeting to direct transposition into MGEs, particularly conjugative plasmids, enabling horizontal transfer across bacterial populations [70]. This process is programmable through spacer sequences design and presents features from CRISPR-Cas systems and the canonical Tn7 transposon: (1) PAM dependence: PAM must flank the target DNA sequence and it is variable depending on the CRISPR-Cas system. For example, Type I–F VchCAST (from *Vibrio cholerae* HE-45 CAST) requires 5′–CC–3′ PAM, whereas Type V–K ShCAST (from *Scytonema hofmannii*) necessitates 5′-GGTT-3′ PAM [43,44]; (2) spacer complementarity: the spacer sequence must be complementary to the target site; (3) transposition directionality: RNA-guided integration exhibits directional bias. Most CASTs showed >99 % transposon left-end adjacent to the target, which is similar to the Tn7 transposon [71]; (4) target immunity (see section 2.2). Downstream insertion of the donor is prevalent due to the existing interactions between Cas and Tn7 core proteins (see Fig. 2) [43,44].

### 2.4 Programmable insertion of large DNA fragments with CAST

Although diverse subtypes of Tn7-like CASTs have been identified, only Type I–F and V–K systems have been extensively characterized as platforms for large DNA fragment (>1 kb) insertion in both bacterial and human cells (see Table 1) [31,[72], [73], [74], [75], [76]]. In contrast, Type I-D and Type I–B CASTs mediate smaller payload integration (<1 kb) in *E. coli* by targeting mobilized plasmids or chromosomal DNA [42,68,77].

**Table 1:** The application of CAST, PE-integrase, and nCas9-R2.

Type	Sub-type	Name	Source	Host	Cargo size	Integration Efficiency	Summary	Reference
CAST	I–F	VchCAST	*Vibrio cholerae*	*E. coli*	>5 kb	<5 %	Pros: V–K CAST's minimal system (Cas12k, TnsA, TnsC, TnsB) offers superior engineering flexibility than I–F CAST system. Cons: V–K CAST has higher co-integration byproducts than I–F CAST. In both CAST systems, the RE/LE scars are generated at the target site.	[43]
	I–F	VchCAST	*Vibrio cholerae*	*Corynebacterium glutamicum*	9 kb	13.4 %		[76]
	I–F	eCAST-2 (PseCAST)	*Pseudoalteromonas*	Mammalian cells	>10 kb	<0.5 %		[87]
	I–F	EvoCAST (PseCAST)	*Pseudoalteromonas*	Mammalian cells	14.78 kb	∼10 %		[31]
	I–F	INTEGRATE (VchCAST)	*Vibrio cholerae*	*E. coli*	0.98–10 kb	>99 %		[45]
	V–K	ShCAST	*Scytonema hofmannii*	*E. coli*	2.2–10 kb	25 %		[44]
	V–K	ShHELIX	*Scytonema hofmannii*	*E. coli*	9.8 kb	up to 80 %		[44]
	V–K	N_7_HELIX	*Nostoc* sp. PCC7101	Mammalian cells	2.6 kb	<0.06 % (S15)		[46]
	V–K	MG64-1	V–K CAST homology was identified from a metagenomic gene	Mammalian cells	3.2-kb	∼1 %		[98]
PE-integrase		TwinPE-Bxb1	Human codon-optimized Bxb1	Mammalian cells	5.6 kb	12–17 %	Pros: PE-integrase enables RNA-guided integration of large DNA cargos in mammalian cells with higher efficiency than CAST or nCas9-R2 systems. Cons: PE-integrase has a large molecular size, which complicates delivery and results in an *attL/attP* scar after integration.	[19]
		TwinPE-evoBxb1	evoBxb1 was obtained from PACE evolution	Mammalian cells	1.7–10.5 kb	>20 %		[114]
		TwinPE-eeBxb1	eeBxb1 was obtained from PACE evolution	Mammalian cells	10.5 kb	35 %		[114]
		PASTE		Mammalian cells	36 kb	>10 %		[112,116]
		PrimeRoot.v2-Cre		Plant	11.1 kb	∼1 %		[113,118]
		PrimeRoot.v3-FLP		Plant	4.9 kb	6.3 %		[113,118]
nCas9-R2		STITCHR	*Bombyx mori*	Mammalian cells	0.7–12.7 kb	5∼10 %	Pros: nCas9-R2 enables truly scarless genomic insertion, unlike CAST or PE-integrase systems. Cons: nCas9-R2 suffers from low integration efficiency and a large fusion protein size that complicates delivery.	[133]

Note: the symbol (S15) denotes the requirement of cofactor S15 [46].

#### 2.4.1 Large DNA fragment insertion using type I–F CAST

The characterization and application of Type I–F CAST, spanning from bacterial to human cells, have progressed through several distinct stages: the initial stages focused on *E. coli* optimization, while subsequent stages were concentrated on human cell applications. Klompe et al. (2019) reconstituted VchCAST in *E. coli* BL21(DE3) with three engineered plasmids: (1) pDonor, encoding a minimal transposon (∼1 kb) flanked by TnsB-recognized LE/RE that served as the insertion cargo sites [78,79]; (2) pQCascade, expressing Type I–F CRISPR-Cas subunits (Cas5/6/7/8), TnsQ, and a CRISPR array targeting *lacZ* with the canonical 5′–CC–3′ PAM [66,80]; (3) pTnsABC, harboring three core transposon genes (tnsA, tnsB, and tnsC). Co-expression of the three plasmids generated a programmable RNA-guided transposon complex that inserted the donor cargo into the *lacZ* target site. PCR analysis revealed insertions 48–50 bp downstream of the 32-nt spacer-binding site, exhibiting undirected orientation. Cargo size dependency assays (from 0.29 to 10.1 kb) showed a peak in integration efficiency at 775 bp (∼25 %), with substantial reduction for constructs <0.4 kb or >2 kb (efficiency became <5 % for cargo >5 kb and was minimal at 10.1 kb [43]).

Building upon prior multi-plasmid systems, Vo et al. (2021) engineered a consolidated vector platform—pSPIN—expressing all VchCAST components as a polycistron in *E. coli* (i.e., under a unique transcriptional control). This single-vector system, termed “INTEGRATE”, exhibited a significantly enhanced RNA-guided integration efficiency. The pBBR1 backbone carrying the constitutive promoter J23119 achieved ∼100 % efficiency for 0.98-kb cargo at 25 °C [81], which represented a dramatic improvement over previous tool. Crucially, INTEGRATE maintained near complete (>99 %) genomic insertion efficacy across cargo sizes (0.98–10 kb) when cultured at 30 °C. Furthermore, INTEGRATE eliminated undirected insertions by enforcing a strict directional bias (right-end downstream of the target), which enhanced product purity. These advances establish INTEGRATE as a high-fidelity platform for effective, large-payload DNA integration [45].

Two VchCAST orthologs from *Gammaproteobacteria*—Tn7476 and Tn7472—were evaluated in *E. coli* for large DNA integration (10 kb). Although phylogenetically distinct from VchCAST, comparative analyses showed neither variant exceeded VchCAST insertion efficiency [66]. VchCAST demonstrated cross-species functionality: it achieved 3.64 % integration efficiency (<1 kb) in *Bacillus subtilis*, and 13.4 % in *Corynebacterium glutamicum* with cargo sizes up to 9 kb [76].

Lampe et al. (2024) adapted VchCAST for large DNA integration in human cells. To address the absence of eukaryotic polycistronic sequences, they engineered combinatorial fusions of VchCAST components with nuclear localization signals (NLSs), epitope tags, and transcriptional activation domains (VPR or VP64) [[82], [83], [84]], using transcriptional activation strength as a functional proxy to screen for optimal assembly configurations. In the resulting eCAST-1 system—where TnsA and TnsB were linked via a bipartite NLS while other components were expressed separately—plasmid-to-plasmid integration of a chloramphenicol resistance gene (*CmR*) donor was confirmed by Sanger sequencing, nested PCR, and TaqMan qPCR. These assays revealed a cargo insertion 49 bp downstream of the spacer-binding site with undirected orientation. However, the efficiency was critically low (<0.1 %) on the plasmid target. To improve the performance, they reconstructed an integrase (eCAST-2.1) using the *Pseudoalteromonas* CAST (PseCAST) identified through bioinformatic mining and experimental validation. eCAST-2.1 achieved 0.4 % integration efficiency—a 40-fold increase over eCAST-1. Further optimization of crRNA sequence, NLS placement, and plasmid amount generated eCAST-2.2, yielding 3–5 % efficiency on plasmid integration, with payloads >10 kb that remained inefficiently integrated (<0.5 %). By incorporating an *E. coli*-derived co-effector ClpX—an essential sequence-specific AAA^+^ ATPase that regulates the folding of substrate proteins [85,86]—into eCAST-2.2, they created eCAST-3 and achieved ∼1 % integration efficiency on genomic loci. This improvement came, however, at the cost of significant cytotoxicity in human cells [87].

To overcome low integration efficiency in genomic loci and ClpX cytotoxicity, the team further engineered multiple PseCAST variants through structure-guided mutagenesis of CAST components. They identified a Cas8 variant (R241K/A244S) that conferred a 3.5-fold enhancement in integration efficiency over wild-type PseCAST [77]. In a parallel effort, Witte et al. developed EvoCAST via phage-assisted continuous evolution (PACE) and rational design [88]. They integrated a TnsB variant containing 10 mutations into PseCAST to eliminate ClpX dependency in human cells. This evolved system achieved 10–30 % integration efficiencies across 14 diverse genomic loci in human cells, representing >400-fold enhancement over wild-type PseCAST. Crucially, EvoCAST efficiently integrated large cargoes—with an efficiency close to 10 % at 14.78 kb—and delivered therapeutic payloads to clinically relevant targets, including safe harbors, cancer immunotherapy sites [31].

#### 2.4.2 Large-DNA-fragment insertion using type V–K CAST

Similar to Type I–F CAST, the compact V–K CAST system—comprising only four core components (TnsB, TnsC, TnsQ, and Cas12k)—has been explored in both bacterial and mammalian cells. The initial work by Feng Zhang's group (2019) achieved an RNA-guided integration of 2.2-kb cargos into *E. coli* plasmids using V–K CAST systems (ShCAST, and AcCAST, the latter from *Anabaena cylindrica*) expressed from a single plasmid (pHelper). These systems mediated unidirectional insertion downstream of a 5′-GTN PAM, with distinct PAM-to-LE distances: 60–66 bp for ShCAST, 49–56 bp for AcCAST. Later characterization of ShCAST revealed cargo size-dependent efficiency in plasmid-to-plasmid integration assays: 75 % for 0.5-kb inserts, decreasing to 25 % for 2.2–10 kb cargos [44,73,89].

Despite high efficiency in *E. coli*, V–K CAST specificity is compromised by TnsA deficiency, which causes cointegrate products [40,44]. Additionally, the uncontrolled DNA-binding capacity of TnsC—uncoupled from CRISPR targeting—drives accidental insertions in wrong locations and creates inconsistent outcomes, thereby failing to meet the precision and reliability standards required for therapeutic genome editing [[90], [91], [92]]. Tou et al. (2023) engineered ShHELIX by fusing the sequence-specific DNA nickase nAniI to the N-terminus of ShCAST's TnsB [93], which retained on-target efficiency while reducing cointegrates 39-fold (plasmid-to-plasmid integration assay) and 16-fold (genome target). Diverse cargo sizes (from 2.1 to 9.8 kb) pointed out that ShHELIX maintained high integration efficiency, achieving up to 80 % even for 9.8-kb payloads. Moreover, the HELIX engineering strategy was successfully extended to ShCAST homologs (AcCAST and ShoCAST, which was also derived from *Scytonema hofmannii*), confirming its broad generality. To enhance integration purity, TnsC confinement—obtained by tethering it to Cas12k—was implemented in V–K variants and determined a reduction in TnsC diffusion radius. This spatial restriction localized the assembly of transposons to CRISPR-specified genomic sites, which increased, in *E. coli*, on-target efficiency to 97.5 % and specificity from 88.4 % to 96.5 %. Implementing this system in mammalian cells required making use of N_7_CAST (from *Nostoc* sp. PCC7101) and its derivative N_7_HELIX, as ShHELIX/AcHELIX failed to work. High-throughput sequencing verified targeted insertions by both systems in plasmid integration assay, with N_7_HELIX reducing plasmid cointegrate formation from 41.9 % to 7.9 %. This improvement was not observed in genomic integration assays. Inspired by the co-factor ClpX dependence observed in Type I–F CAST system, prokaryotic ribosomal S15 was introduced to fulfill analogous requirements for VchCAST complex assembly [92,94]. This intervention yielded detectable but minimal integration efficiency (<0.06 %) with 2.6-kb cargos in human cells [46,89].

A metagenomically mined V–K CAST homology (MG64-1) was engineered through comprehensive optimization in order to improve the low genomic integration efficiency of V–K CAST in human cells. Researchers developed an all-in-one plasmid system by fusing all components of the MG64-1 system to nuclear localization signals and functional accessory domains—including Sso7d, H1core, HMGN1, and ClpX [[95], [96], [97]]—resulting in ∼1 % stable integration efficiency of 3.2-kb therapeutic cargos, a significant improvement over the ShCAST systems in mammalian cells [98].

#### 2.4.3 DNA fragment insertion using type I–B and I-D CAST

Although Type I–F and V–K CASTs have been extensively characterized, their Type I–B and I-D counterparts remain relatively underexplored and their functionality is currently confined to prokaryotic hosts. Pioneering work by Saito et al. established that Type I–B systems AvCAST (I–B1) and PmcCAST (I–B2) can be harnessed for RNA-guided 0.5-kb cargo integration in *E. coli* in a plasmid-to-plasmid context (only AvCAST achieved genomic integration) while operating under distinct PAM requirements—5′-AT for AvCAST, 5′-ATG for PmcCAST. These Type I–B variants exhibited divergent directional biases despite proximal insertion loci: AvCAST integrated payloads 43–49 bp downstream of the spacer-binding site with its RE proximal to PAM, whereas PmcCAST positioned a cargo 48–57 bp downstream of the spacer 3′-terminus with its LE near PAM [41]. Parallel studies on Type I-D systems CyCAST and McCAST (derived from *Myxacorys californica* WJT36-NPBG1) revealed conserved mechanistic frameworks featuring obligate 5′-GTT PAM recognition [[99], [100], [101]], unidirectional LE-cargo-RE orientation, and defined insertion windows—with kanamycin as cargo—at 69–81 bp post PAM for McCAST and 70–80 bp downstream of the protospacer for CyCAST under identical PAM conditions [42,68].

Crucially, as detailed in Section 2.3, the native dual transposition modes of I–B/I-D CASTs mediated by TnsQ-family proteins enable strategic engineering through TnsD deletion, which enhances TnsQ-driven integration to boost efficiency by 2.5-fold in CyCAST and 3.0-fold in AvCAST [41,42].

## 3 PEs

**Fig. 3:**
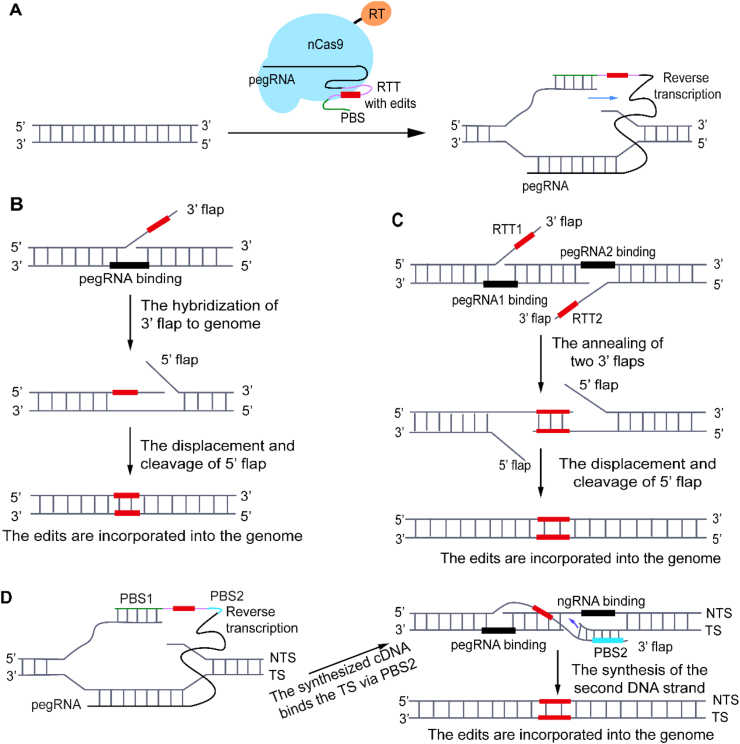
The working mechanism of PE and its engineered variants. (**A**) The nCas9 (H840A) protein fused to an RT (e.g., M-MLV) forms a ribonucleoprotein complex with a pegRNA, which contains a PBS and an RTT encoding the desired edit (the red rectangle). After binding the target locus, the complex nicks the NTS, allowing PBS hybridization and the prime RT-mediated synthesis of the edited DNA strand. (**B**) The resulting 3′ flap, which contains the newly synthesized edited sequence, is incorporated into the genomic site upon 5′ flap displacement, excision, and ligation. (**C**) In a dual-pegRNA strategy, a second pegRNA (pegRNA2) bearing an RTT (RTT2) complementary to RTT1, binds to the opposite strand and facilitates the synthesis of its edited part. This enables a double-stranded DNA generation, due to the annealing of the products from RTT1 and RTT2, which is incorporated into the genome after the removal of the 5′ flaps. (**D**) The TJ-PE system employs a single pegRNA that contains two PBS sequences. PBS1 hybridizes to the NTS to initiate the first-strand DNA synthesis. Then, the cDNA product from PBS2 (blue rectangle) hybridizes to the TS and the first-strand DNA serves as a template for the second-strand DNA synthesis. The resulting double-stranded DNA edit is integrated into the genome through cellular DNA repair mechanisms.

PE was first established in 2019 and has since evolved through seven generations, from PE1 to PE7. The early systems PE1 to PE3 rely on the RT from Moloney murine leukemia virus (M-MLV), with PE2 incorporating an engineered RT variant that enhances editing efficiency, and PE3 that introduces an additional sgRNA to nick the non-edited strand for further improvements [21]. Subsequent versions, PE4 and PE5, were developed by co-expressing a dominant mutant of MLH1 protein (MLH1dn: MLH1Δ754–756) to suppress DNA mismatch repair (MMR) and increase editing efficiency and product purity in combination with PE2 and PE3 systems, respectively [102]. PE6 employs a novel RT domain that was engineered through PACE and offered improved catalytic properties compared to the M-MLV variant [103]. PE7, which was created last year, further improved editing efficiency by leveraging the eukaryotic La protein to increase pegRNA stability [104]. The core mechanism shared across all the PE systems involves a PE ribonucleoprotein (RNP) complex that binds genomic DNA, where nCas9 cleaves the NTS to expose a DNA gap. Then, the pegRNA hybridizes to this site via its PBS sequence, allowing the RT domain to synthesize an edited DNA 3′ flap using the RTT sequence (see Fig. 3A). This 3′ flap subsequently invades the duplex and displaces the original 5′ flap, which would be otherwise cleaved by a structure-specific endonuclease (e.g., FEN1) or removed by a 5′ exonuclease (e.g., EXO1) [21,105,106]. Without the 5′ flap, the desired 3′ flap is incorporated and repaired using cellular DNA machinery (see Fig. 3B). A key advantage of PE is the avoidance of DSBs, which enables highly precise genome editing.

### 3.1 PE variant enables short DNA fragment insertion (<1 kb)

A major limitation of conventional PE lies in the very limited capacity of inserting large DNA fragments, typically not exceeding 50 bp [21]. To overcome it, several PE variants were developed by leveraging dual pegRNAs. The dual-pegRNA-based approach employs two pegRNAs targeting opposite DNA strands with RTTs containing complementary sequences. After reverse transcription, the resulting single-stranded DNA products anneal to form a double-stranded intermediate, which is then integrated into the genome via 5′ flap cleavage and endogenous repair pathways (see Fig. 3C). This significantly improves the efficiency of long-fragment insertions, beyond the limits of the standard PE2/PE3 systems [19,[107], [108], [109]]. Zhuang et al. (2022) reported a HOPE system that achieved 32.6 % efficiency for inserting a 24-bp Flag-tag at the *HEK* site3 locus, outperforming PE2 and PE3 (12.7 % and 19.6 %, respectively) [107]. Similarly, TwinPE—developed by David Liu's group—showed over 50 % efficiency in the insertion of a 38-bp *attB* or a 50-bp *attP* site at a safe harbor locus in mammalian cells. It also accomplished a 108-bp insertion with 16 % efficiency—by far exceeding the 0.8 % efficiency reached with PE3 [19]. The GRAND editing system, engineered by Yin's group, enabled the insertion of 150-bp and 250-bp fragments at efficiencies of 63 % and 28.4 %, respectively. Furthermore, GRAND permitted the integration of a 150-bp sequence in five endogenous genomic loci with efficiencies ranging from 12.0 % to 42.4 %, which exceeded the performance of PE3 (below 2.2 % at every site) [108].

Besides the dual-pegRNA approach, Zheng et al. (2023) developed the TJ-PE system that achieved longer DNA insertions by using only a single pegRNA. Here, the innovation lies in the inclusion of a second PBS (PBS2) upstream of the RTT domain. During the initial reverse transcription, the sequence including the edit and the PBS2 is copied into a single-stranded cDNA flap. Then, PBS2 hybridizes to the target strand immediately downstream of the nick site that resulted from the action of a nicking sgRNA (ngRNA—see Fig. 3D). This hybridization event facilitates the second-strand synthesis, which uses the cDNA as a template, thereby generating a double-stranded DNA product *in situ*. With this innovative mechanism, TJ-PE accomplished insertion efficiencies from 11.4 % to 50.5 % on larger fragments (200–500 bp) in HEK293T cells, corresponding to 19- up to 35-fold improvements over conventional PE3 systems [110].

### 3.2 Large DNA fragment insertion using PE-integrase

**Fig. 4:**
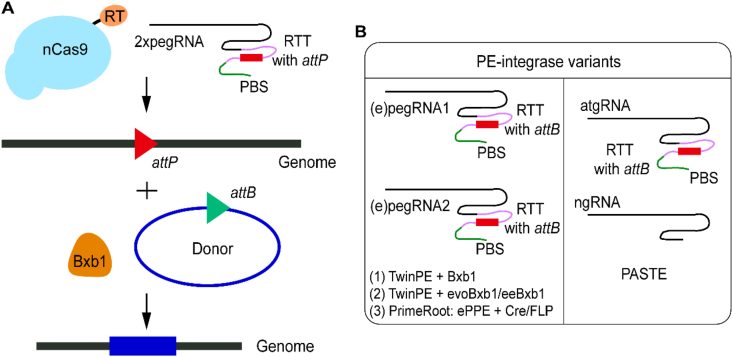
The integration mechanism and variants of the PE-integrase system. (**A**) Strategy for targeted cargo insertion using PE-integrase. An nCas9-fused RT first introduces short *attP* sites into the genome via a dual-pegRNA system, wherein each pegRNA encodes an *attP* sequence within the RTT. The integrase Bxb1 then catalyzes the integration of a donor cassette carrying *attB* sites. (**B**) Engineered PE-integrase variants developed through directed evolution and system redesign. Systems (1), (2), and (3) employ dual-pegRNA or dual-enhanced-pegRNA (epegRNA) designs, whereas the PASTE system uses a single atgRNA (containing an *attB*-encoding RTT) and an ngRNA. Systems (1) and (2), developed by David Liu's lab, were optimized for mammalian cells; (3) PrimeRoot was designed for plant genome editing.

Although dual-pegRNA systems significantly improved the efficiency of prime editing for medium-sized inserts, their performance remains limited for large DNA fragments exceeding 400 bp. The GRAND system exhibited an insertion efficiency below 0.38 % for fragments longer than 400 bp, which further declined to 0.002 % for a 1085-bp fragment [108]. To overcome this limit, researchers have integrated site-specific recombinases—such as the serine integrase Bxb1 [111]—into PE platforms [19,[112], [113], [114], [115]]. In this hybrid approach, a PE variant is first used to insert an *attP* or *attB* recombination site into a genomic locus. Subsequent co-expression of the Bxb1 recombinase and a donor template carrying the complementary *att* site (e.g., *attB* if the genome contains *attP*) enables Bxb1-mediated targeted integration of the donor cassette without inducing DSB (see Fig. 4A) [19]. The combination of TwinPE with a human codon-optimized Bxb1 achieved 12–17 % insertion efficiency for a 5.6 kb cargo at the *CCR5* locus in HEK293T cells [19]. David Liu's group employed PACE to obtain the enhanced variants evoBxb1 (V74A) and eeBxb1 (V74A-E229K–V375I). These mutants accomplished insertion efficiencies of 9.5 % and 20 %, respectively, on a 5.6 kb sequence at the *Rosa26* safe-harbor locus in N2A cells, whereas the wild-type Bxb1 arrived only at 3.2 %. Moreover, when tested with cargo sizes ranging from 1.7 to 10.5 kb, the evolved integrases retained efficiencies above 20 %, whereas the wild-type version dropped below 10 % for inserts larger than 5.6 kb. Notably, eeBxb1 achieved 35 % integration efficiency for a 10.5 kb fragment at the *CCR5* site, representing a 3.8-fold improvement over the wild-type system [114].

In addition, an alternative strategy named PASTE was developed for large DNA integration using a single-pegRNA design. PASTE employs an integrase and a prime editor to enable programmable large-fragment insertion [112,116]. The initial version, PASTEv1, comprises a PE expression vector, a donor vector co-expressing Bxb1 and the cargo, an ngRNA, and an attachment site-containing pegRNA (atgRNA) with an *attB* sequence embedded in the RTT (see Fig. 4B). This configuration allowed ∼15 % insertion efficiency for a 969 bp cargo. PASTEv2 improved this efficiency up to 30 % by fusing Bxb1 to the reverse transcriptase domain via a (GGS)_6_ linker. Further optimization led to PASTEv3, which incorporated a stabilized atgRNAv2 through scaffold engineering [117]. PASTEv3 demonstrated robust insertion across multiple genomic loci and cargo sizes, exceeding 10 % efficiency for a 36 kb insertion at the *ACTB* locus in human cells [112].

The development of PE systems for large DNA fragment insertion has interested also plant genome engineering. A remarkable advancement in this area is the PrimeRoot system, developed in 2023, which combines dual epegRNAs, an enhanced plant prime editor (ePPE), and the activity of a site-specific recombinase such as Cre or FLP (see Fig. 4A) [113,[118], [119], [120], [121]]. This system evolved through three iterative generations, each offering distinct improvements. In PrimeRoot.v1, the recombinase was expressed independently of the ePPE [122] component. PrimeRoot.v2, in contrast, featured a redesigned architecture where the recombinase was fused to the C-terminal end of ePPE via an SV40 NLS and a flexible peptide linker, raising the coordination between the editing and the recombination steps. When inserting a 720-bp cargo in rice protoplasts, PrimeRoot.v2 coupled with Cre recombinase (PrimeRoot.v2-Cre) attained a precise insertion efficiency of 6 % at the target locus, outperforming PrimeRoot.v1 by 3-fold. More importantly, PrimeRoot.v2-Cre maintained detectable activity (∼1 %) in integrating exceptionally large sequences, up to 11.1 kb, across multiple endogenous genomic sites in rice [113]. To further augment performance, PrimeRoot.v3 incorporated a sequential transformation strategy: the first transformation introduced the PrimeRoot components to place the desired recombinase site (RS) into the rice callus genome, and a second transformation delivered the donor vector containing the large cargo. Using an optimized FRT1 variant (F1m2) as the recognition site for FLP recombinase, PrimeRoot.v3-FLP scored an insertion efficiency of 6.3 % for a 4.9-kb cargo, marking a 2.4-fold enhancement over single-step transformation protocols [113]. This progression highlights how rational protein engineering and sophisticated delivery methods could synergize to overcome historical barriers to large DNA integration in plants.

## 4 The TPRT mechanism of R2 retrotransposon

**Fig. 5:**
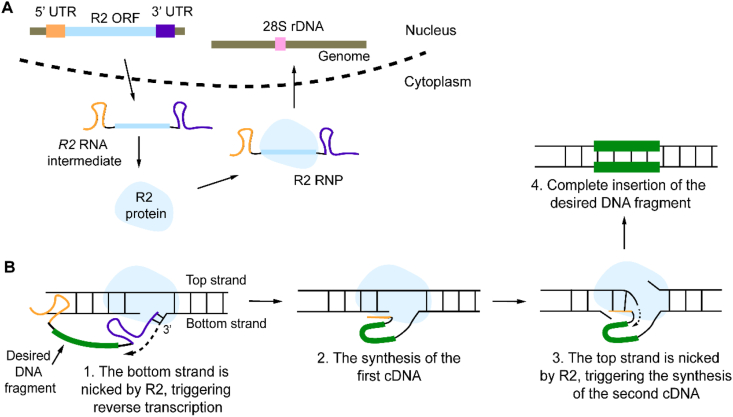
The complete TPRT process of the R2 retrotransposon. (**A**) The R2 open reading frame (ORF) is transcribed into a full-length R2 RNA intermediate containing 5′ and 3′ UTRs. Following the export to the cytoplasm, the RNA is translated into the R2 protein, which binds its own RNA to form a ribonucleoprotein (RNP) complex. This complex re-enters the nucleus and targets 28S rDNA specifically. (**B**) The details of TPRT. First, cleavage of the bottom DNA strand by the endonuclease domain of R2 generates a 3′-OH, which serves as a primer for reverse transcription (initiated at the 3′ UTR of the R2 RNA). The newly synthesized cDNA is then incorporated into the genomic site through strand displacement. Subsequently, the R2 protein nicks the top strand to start second-strand DNA synthesis. The resulting double-stranded DNA product is ultimately integrated into the genome via the host DNA repair pathways.

A distinct approach leverages eukaryotic non-long terminal repeat (non-LTR) retrotransposons, such as the R2 element, to carry out insertion via target-primed reverse transcription (TPRT) that entirely avoids generating DSBs [123,124]. The R2 system comprises two essential components: the R2 protein and the R2 mRNA flanked by synthetic 5′ and 3′ untranslated regions (UTRs) that are critical for the ordered cleavage process. The R2 protein contains several functional domains, including DNA-binding motifs, an RNA-binding domain (RBD), a RT domain, and an endonuclease domain [125,126]. TPRT process begins with the transcription of genomic R2 DNA into a full-length RNA intermediate containing defined 5′ and 3′ UTRs (see Fig. 5A). This intermediate associates with the same R2 proteins in the cytoplasm, forming a stable R2 ribonucleoprotein (RNP) complex. Upon nuclear import, the RNP specifically targets the 28S ribosomal DNA (rDNA) locus [123,124], where the R2 protein introduces a single-strand nick in the target strand (also referred to as bottom strand), generating a free 3′-hydroxyl group (see Fig. 5B). The exposed 3′-OH end subsequently hybridizes with the 3′ terminus of the RNA intermediate [127], which acts as a template for reverse transcription. Primed by the DNA 3′-OH, reverse transcription leads the synthesis of a complementary DNA (cDNA) strand [123,128,129]. According to the proposed models, following the first-strand cDNA synthesis, the top strand of the target DNA is cleaved, and the nascent cDNA serves as a template for the second-strand DNA synthesis [130]. Integration is finalized through the repair of the fresh DNA intermediates by the endogenous cellular machinery [129].

A key advantage of the TPRT mechanism is its sequential DNA cleavage strategy, which circumvents DSB generation and enhances targeting specificity. Structural insights from cryogenic electron microscopy (cryo-EM) and biochemical experimental studies have led to a model in which the UTRs play regulatory roles in this process [125,131]. Specifically, the 3′ UTR facilitates bottom-strand cleavage through interactions with the RBD of the R2 protein, while the 5′ UTR is involved in subsequent top-strand excision. Owing to a higher binding affinity for the RBD, the 3′ UTR effectively outcompetes the 5′ UTR, temporarily suppressing top-strand cleavage activity [125]. However, during elongation of reverse transcription, the structural integrity of the 3′ UTR is disrupted, leading to its dissociation from R2 [125]. This release renders the RBD accessible for 5’ UTR, thereby enabling cleavage of the top strand and facilitating complete integration of the cDNA strand [125].

### 4.1 Specific integration at 28S rDNA using R2

Although the molecular details of sequential DNA cleavage during R2 retrotransposon integration have not been fully elucidated [125,131], its fundamental mechanism, TPRT, has been exploited for targeted DNA insertion. This approach involves replacing the native R2 coding sequence in the RNA intermediate with a desired DNA cargo fragment. In 2020, Fujiwara's group demonstrated the application of R2Ol, derived from the medaka fish (*Oryzias latipes*), to efficient transgene integration into the 28S rDNA locus in zebrafish [132]. They injected into the cells an engineered R2Ol mRNA construct comprising a 5′ UTR (265 bp), the R2Ol transcript (3821 bp), and a 3′ UTR (108 bp). Their findings highlighted that a 100-bp homology arm (HA) between the 3′ UTR and the target site significantly enhanced insertion efficiency, elevating it from 12 % to 96 %, compared to a 4-bp annealing sequence. Furthermore, leveraging the TPRT mechanism, the team successfully inserted an enhanced green fluorescent protein (EGFP) expression cassette (including promoter, EGFP coding sequence, and terminator) flanked by the 5′ and 3′ UTRs of R2Ol. This strategy achieved up to 95 % insertion efficiency in the zebrafish F0 generation. A study published last year utilized R2Tg, from *Taeniopygia guttata*, to insert a GFP cargo into human HEK293T cells [129]. This work determined that a minimum of 100 bp homology between the UTRs (both 5′ and 3′) and the 28S rDNA was necessary to achieve high insertion efficiency, approximately 2 %. This study also observed that R2Ol was ineffective in HEK293T cells, underscoring inherent complexity and species-specific barriers that influence retrotransposition efficiency in mammalian cells. The authors also developed an enhanced en-R2Tg system [129]. This optimized system featured a truncated 3′ UTR (a deletion from +1 to +244) and a refined R2Tg protein based on an AI-guided protein engineering approach. The modifications proved highly effective: delivery of en-R2Tg mRNA and donor RNA into mouse embryos resulted in GFP insertion efficiencies up to 60 %, with exceptional target specificity exceeding 99 % at the 28S rDNA locus, demonstrating significant potential for precise genetic engineering applications [129].

### 4.2 Programmable large DNA integration using the nCas9-R2 system

**Fig. 6:**
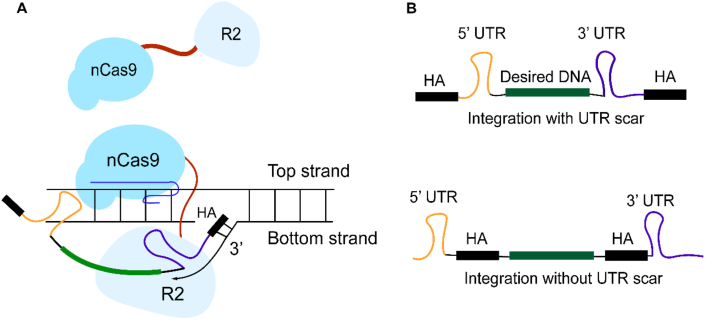
The programmable genomic integration using a nCas9-R2 fusion system. (**A**) Working mechanism of the nCas9-R2 fusion protein. The nCas9:sgRNA complex directs the R2 RNP to a target genomic site. nCas9 nicks the NTS to expose a 3′-OH end that anneals to the HA from the RNA intermediate to initiate reverse transcription. (**B**) Design strategies for scarred and scarless integration. In the scarred design, the two HAs are located outside the UTR regions. In the scarless design, both HAs are positioned internally, directly flanking the DNA fragment of interest, enabling reverse transcription and precise insertion without extraneous sequences.

Although the R2 system enables precise and highly efficient integration in mammalian cells, its exclusive targeting of the 28S rDNA locus constrains a broader applicability in clinical therapy due to limited programmability. To achieve the retarget of R2, several researches have explored strategies by fusing the R2 protein with a *Streptococcus pyogenes* Cas9 nickase (nSpCas9; H840A variant—see Fig. 6A) [131,133]. In such designs, the nSpCas9:sgRNA complex serves to guide the R2 machinery to specific DNA sequences and facilitates TPRT. A study carried out by Feng Zhang and colleagues (2023) pointed out that a nSpCas9–R2Bm (from *Bombyx mori*) fusion protein, in conjunction with a sgRNA targeting a sequence from *Drosophila virilis*, could direct, in an *in vitro* assay, the insertion of a cassette containing a *CMV* promoter followed by the 3’ UTR of R2Bm and a 10-nt HA adjacent to the sgRNA nick site [131]. Gootenberg et al. (2025) developed a scarless programmable integration tool named STITCHR, which employs an engineered nSpCas9–R2Tocc^Δ1–169^ fusion (derived from contaminants of *Talpa occidentalis*) [133]. By designing sgRNAs targeting the *AAVS1* locus and placing the HAs between the two UTRs (see Fig. 6B), the system achieved up to 10 % precise integration of the Gaussia luciferase exon 2 *in vitro*, compared to 1.5 % obtained in the absence of the nSpCas9:sgRNA complex. This scarless integration strategy was successfully transferred to mammalian cells. The STITCHR system shows versatility for retargeting multiple genomic loci—including *AAVS1*, *NOLC1*, *LMNB1*, and *EMX1*—with integration efficiencies ranging from 5 % to 11 % using either single or paired sgRNAs. Integration efficiency appears largely independent of the payload size. As insertions ranged from 0.7 kb to 12.7 kb into the *NOLC1* locus, efficiency was always around 5–10 %, with the largest payload of 12.7 kb still achieving near 10 % precise integration [133].

## 5 Challenge, perspective and conclusion

Despite considerable advances in large DNA cargo (>1 kb) insertion technologies—such as the CAST, PE-integrase, and R2 retrotransposon systems—their transition into clinical applications remains deeply challenging. A critical drawback is the low integration efficiency in mammalian cells (see Table 1) largely attributable to their reliance on prokaryotic-derived cofactors and the poorly characterized integration structural mechanisms [46,87,125]. Furthermore, the inherently large and multi-component architecture of these systems—for instance, the four core proteins in the minimal Type V–K CAST system, the SpCas9-RT-recombinase complex in PE-integrase, and the bulky nCas9-R2 fusion protein—exceeds the carrying capacity of conventional AAV vectors (<4.7 kb) [134], creating a persistent delivery bottleneck that has not been adequately resolved. Additionally, unintended genomic alterations at the target site, such as the generation of recognition/excision sites (RE/LE) in CAST systems or the residual *attL*/*attP* sequences following Bxb1 serine integrase-mediated recombination [135], continue to pose significant challenges for precise genomic engineering. In contrast, the STITCHR system, which leverages the engineered non-LTR retrotransposon R2, achieves truly scarless integration through rational redesign of its RNA template and HAs, thereby circumventing the incorporation of terminal UTRs into the genome [133]. This approach not only enhances the precision of large DNA cargo insertion but also strengthens its suitability for therapeutic applications. Nonetheless, the development of retrotransposon-based systems for gene-sized integration has advanced more slowly than those employing integrases or CASTs due to the limited understanding of their structural integration mechanisms [125,131].

Current optimization efforts in engineering reverse transcriptase variants or developing split-Cas9-intein systems [103,136,137], have only yielded incremental improvements. While these attempts mitigate to some extent the size constraint through multi-vector delivery strategies, they introduce new complexities: the necessity for precise splitting sites to maintain Cas9 activity, laborious assembly processes, and limited targetability due to the restrictive PAM requirements of SpCas9. These inherent limitations underscore that such modifications are stopgap solutions rather than fundamental breakthroughs.

To truly enable clinical application of large DNA insertion, several tricky challenges should be addressed through some innovative approaches. First, a deeper structural mechanism understanding of DNA integration in mammalian cells is essential. The dependence on bacterial-derived elements not only constrains efficiency but also raises concerns regarding immunogenicity and unintended genomic alterations [87]. Computational modeling and directed evolution approaches could help engineer more efficient and human-compatible CAST or integrase variants with reduced foreign sequence dependency. The integration of immunogenicity criteria—together with protein activity and specificity—in the computational design process could help prevent undesired immune responses *in vivo*. Second, delivery strategies must advance beyond simple vector splitting or protein truncation. While dual- or triple-AAV systems partially alleviate packaging constraints [134,138], they suffer from reduced co-delivery efficiency and increased cellular burden. Emerging technologies—such as engineered synthetic viral vectors (e.g., parvoviruses or hybrid vesicle systems) [139] and non-viral platforms (e.g., virus-like particles or lipid nanoparticles optimized for large cargo) [140,141]—may offer more robust delivery solutions. In particular, LNPs designed for RNP delivery could provide a transient yet efficient means to deliver large editor complexes without exacerbating genomic safety concerns [142,143]. Finally, the exploration of novel systems should extend beyond the currently known compact nucleases. Unbiased mining of microbial and metagenomic databases may reveal previously unknown RNA-guided systems that are both small in size and efficient in human cells [38,39]. Furthermore, structure-guided engineering of existing compact effectors (e.g., Cas12f, TnpB, and IscB) [[144], [145], [146]] could further expand the targeting scope and enhance versatility.

In conclusion, although current systems have established foundational capabilities, their clinical applicability will depend on a paradigm shift—from adapting natural systems to redesigning them with human therapeutic applications. Interdisciplinary efforts combining computational design, protein engineering, directed evolution, and innovative delivery technologies will be essential to accomplish the full potential of large DNA insertion for clinic genetic therapy.

## CRediT authorship contribution statement

**Lifang Yu:** Writing – review & editing, Writing – original draft, Funding acquisition, Conceptualization. **Mario Andrea Marchisio:** Writing – review & editing, Conceptualization.

## Declaration of competing interest

The authors declare that there is no competing financial interests or personal relationships that could have appeared to influence the work reported in this paper.
